# Intraoperative Neuromonitoring: Evaluating the Role of Continuous IONM and IONM Techniques for Emerging Surgical and Percutaneous Procedures

**DOI:** 10.3389/fendo.2022.808107

**Published:** 2022-03-30

**Authors:** Catherine McManus, Jennifer Hong Kuo

**Affiliations:** Department of Surgery, Columbia University Irving Medical Center, New York, NY, United States

**Keywords:** radiofrequency ablation, thyroid, intraoperative nerve monitoring (IONM), recurrent laryngeal nerve, recurrent laryngeal nerve injury

## Abstract

Intraoperative nerve monitoring (IONM) is a tool used during thyroid surgery to assist in the identification of the recurrent laryngeal nerve (RLN). Multiple IONM systems that exist for thyroidectomy require intubation with an endotracheal tube. Given that one of the advantages of thermal ablation procedures, such as radiofrequency ablation, is that they can be done safely without the use of general anesthesia, nerve monitoring systems that utilize cutaneous surface electrodes have been developed, though are not widely available in the United States. This article will review the use of IONM for RFA including the cutaneous surface electrode system.

## Introduction

**Figure 1:**
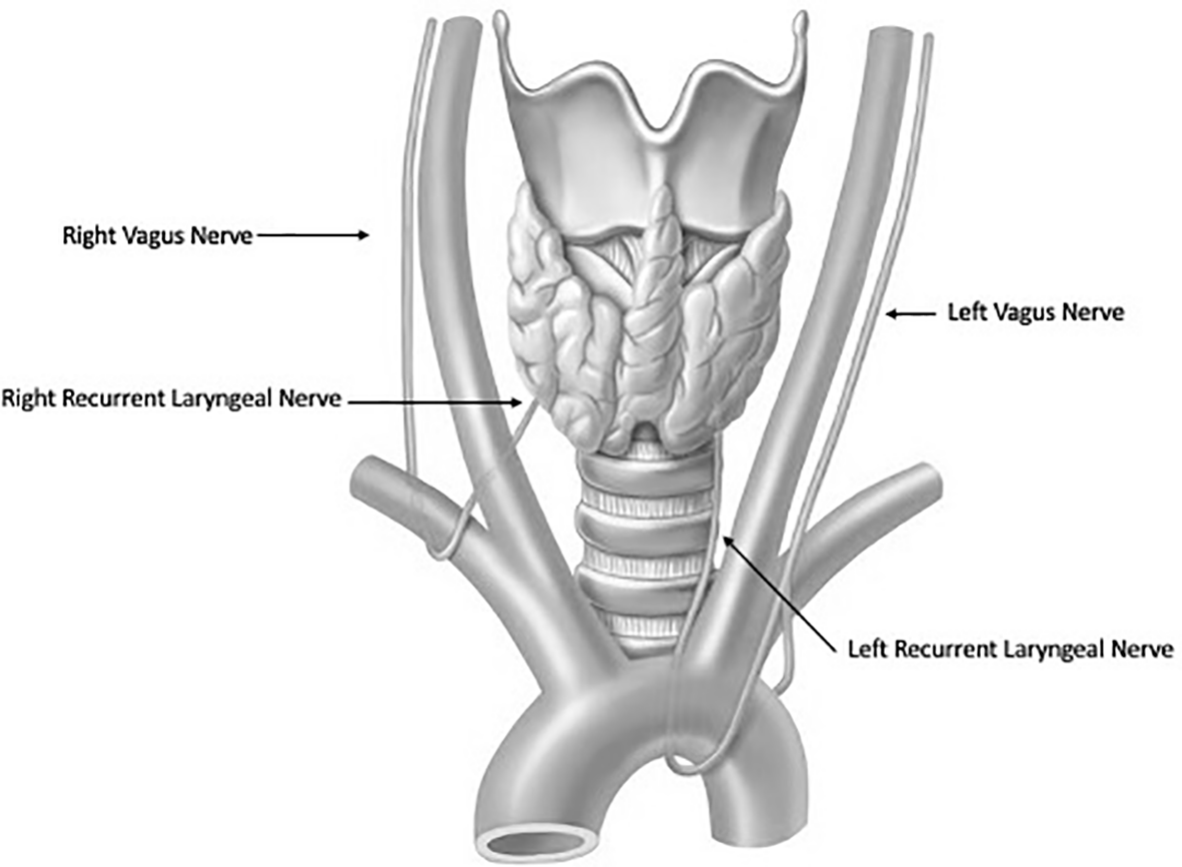
Anatomy of the Recurrent Laryngeal Nerves.

Thyroidectomy is a technically delicate surgery involving highly detailed anatomy. The recurrent laryngeal nerve (RLN) is the most important structure at risk during these operations (**Figure 1**). Traditionally, the gold standard for protecting the nerve is identifying it through careful dissection before proceeding with the removal of the thyroid gland. The importance of definitively identifying the nerve in order to preserve it was highlighted in the 1950s by Riddell and colleagues (1). Since that report, nerve visualization, anatomical knowledge, and surgeons’ experience have been the most important tools for protecting the RLN and still serve as the standard of care. However, despite cautious RLN visual identification, RLN nerve injury can still occur because of anatomical variation, surgeon inexperience, and difficult situations including a large goiter, revision surgery and invasive malignancy. Although the rate of injury to the nerve has been reported to be relatively low (<1%–5%), especially when performed by high-volume surgeons (2, 3) postoperative compromise of voice quality may diminish the patient’s quality of life and trigger litigation for malpractice (4). To help reduce the incidence of dysfunction, intraoperative neural monitoring (IONM) was introduced in the 1970s and has become an increasingly popular adjunct in thyroidectomy (2).

## Different IONM

**Figure 2:**
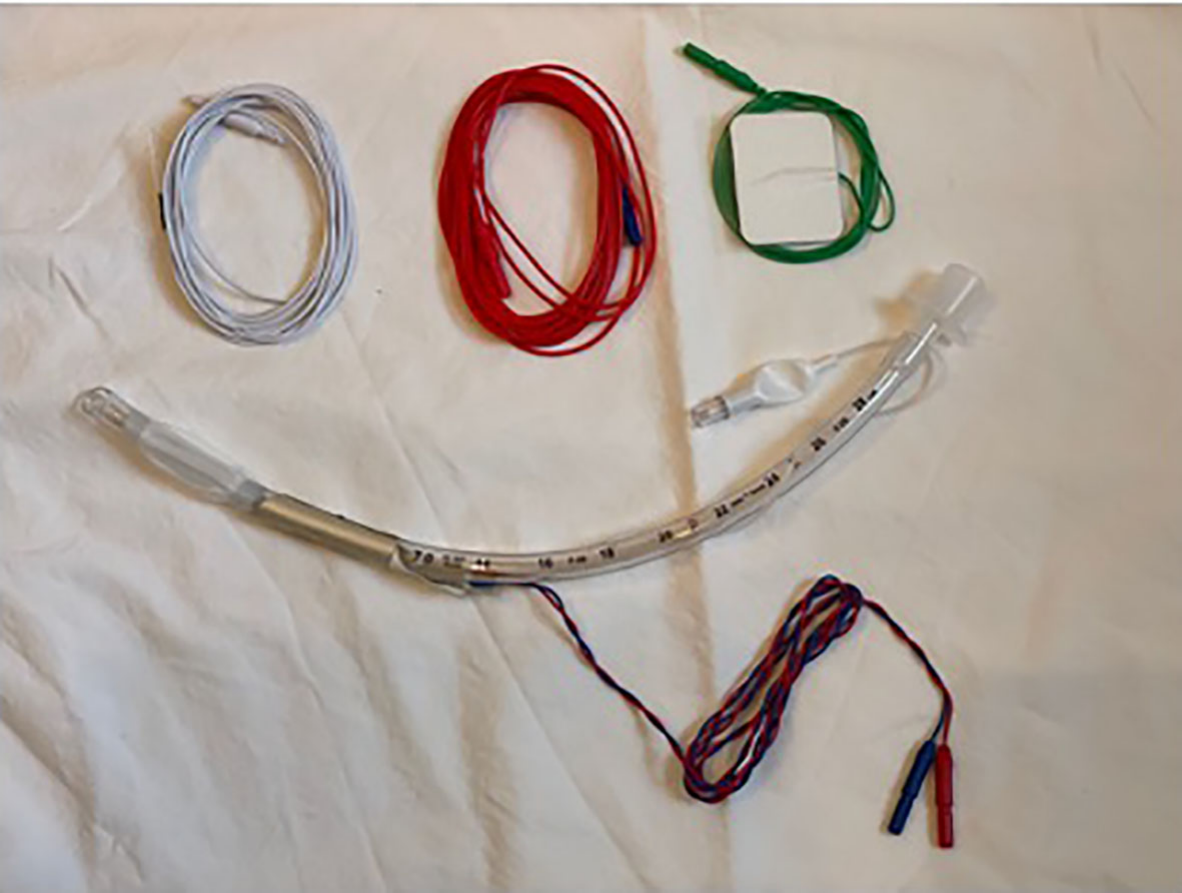
An example of a IONM system. Adhesive electrodes are applied to an endotracheal tube and the blue and red wires are connected to a nerve monitoring system. One needle wire (white) is inserted into the deltoid muscle, a green lead wire sticker is applied to the forehead, and the single red wire connects to a handheld monopolar probe that is used to stimulate the recurrent laryngeal nerve.

The most widely used IONM systems facilitate RLN identification and mapping of the anatomic course of the nerve and uses passive monitoring, in which the presence and quality of the interrogation signal confirms neural integrity, and loss of signal at any point caudal to an injury indicates a nonfunctioning nerve or technical issues. In its first iteration, IONM used needle electrodes inserted through the cricothyroid membrane into the vocal muscle and an intermittent nerve stimulation is conducted with a hand-held monopolar probe that stimulated the RLN, enabling recording the electrophysiological response signal. Currently, most IONM systems involve monopolar hand-held probes that intermittently stimulate the nerve through surface or incorporated electrodes on the endotracheal tube and records the electrophysiological response signal (**Figure 2**). Most systems also allow for continuous nerve stimulation, with a clip electrode mounted on the vagus nerve and surface electrodes affixed to the endotracheal tube recording the electrophysiological response signal (5).

An alternative form of continuous IONM relies on an electromyographic endotracheal tube (EMG-ETT) alone to elicit a laryngeal adductor reflex (LAR), which allows for continuous monitoring of the RLN and VN function (6–8). Irritation to the RLN or VN (i.e. due to traction, compression, heat) results in transient or permanent LAR amplitude declines with permanent LAR loss correlating with postoperative vocal fold paralysis (6, 7). Similar to intraoperative neuromonitoring techniques that use direct nerve stimulation (6, 7), an amplitude decline >50% is considered significant and warrants corrective actions by the surgeon such as cessation of dissection, relaxation of tissues, and warm irrigation (9). The biggest advantage of this technique is the lack of neural electrodes required.

Both intermittent and continuous IONM allow for differentiation between segmental loss of signal (LOS; type 1) and diffuse LOS (type 2), whereas continuous IONM offers provides real-time intraoperative feedback of nerve signals. This facilitates instant detection and release of a distressed nerve can minimize RLN injury. For documentation purposes, the electromyographic recordings can be printed out from the system and filed with the patient’s clinical chart. Both IONM modalities inform the surgical treatment plan and provide strategic direction regarding the need for staged thyroidectomy in the event of definitive LOS on the first side of the operation (9).

## Efficacy of IONM

The efficacy of IONM has been studied extensively, including several large-scale multi-institutional trials, both prospective and retrospective, with conflicting results. There is some evidence to suggest that IONM decreases both transient and permanent RLN injury (10, 11), only transient RLN injury (11, 12), or only permanent RLN injury (13). In contrast, other researchers found no substantial reduction of permanent RLN injury (14), or both transient and permanent RLN injuries (15–17). A 2017 review of 8 meta-analyses confirmed no observable reduction in transient or permanent RLN injury. 740 Routine use of IONM during total thyroidectomy was not found to be cost-effective compared with visual identification alone, though a 2017 study showed that IONM was potentially cost effective in preventing bilateral RLN dysfunction (18).

Although selective use of IONM by many specialties is common in the United States, IONM has become the standard of care in many countries and is endorsed by the German Association of Endocrine Surgeons (19) and the Australian College of Surgeons (20) as a valuable adjunct to RLN visualization.

## Challenges with IONM AND Thermal Ablation

Introduced in the early 2000s, thermal ablation (TA) procedures, such as radiofrequency ablation (RFA) and laser ablation (LA), are increasingly used to treat benign thyroid nodules (21, 22). Short-term studies (<1 to 2 years) showed that TA is effective and safe for the treatment of cosmetic or symptomatic benign nodules, resulting in a reduction in nodule volume of 50% to 80% (23–25). These procedures can be performed under local or general anesthesia. Although RFA appears to be a relatively safe procedure, the potential for injury to the vagus (VN) and recurrent laryngeal nerves (RLN) exists. Similar to RLN injuries incurred during open thyroidectomy, the incidence of thermal injury to either of these nerves during RFA is low, ~1%, but can result in temporary or permanent vocal fold immobility, vocal hoarseness, aspiration and/or dysphagia (26, 27). However, there is limited objective data on the risk of nerve injury during RFA, such as pre and post RFA laryngeal examinations or feedback from a nerve monitor, which has limited the direct comparison of rates of nerve injury with RFA and thyroidectomy.

**Figure 3:**
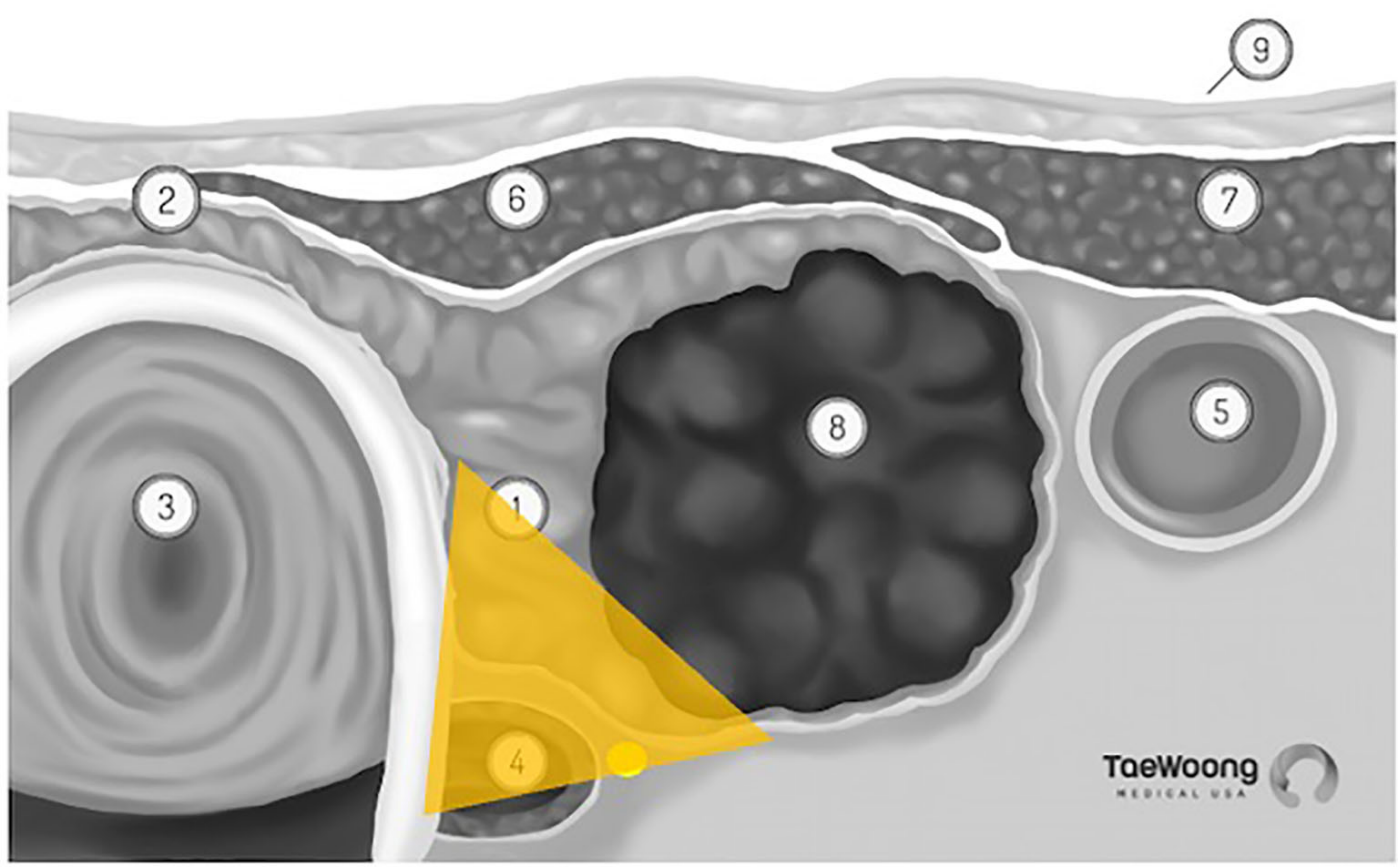
‘Danger Triangle’ housing the recurrent laryngeal nerve (permission to reprint from Taewoong Medical USA).

**Figure 4:**
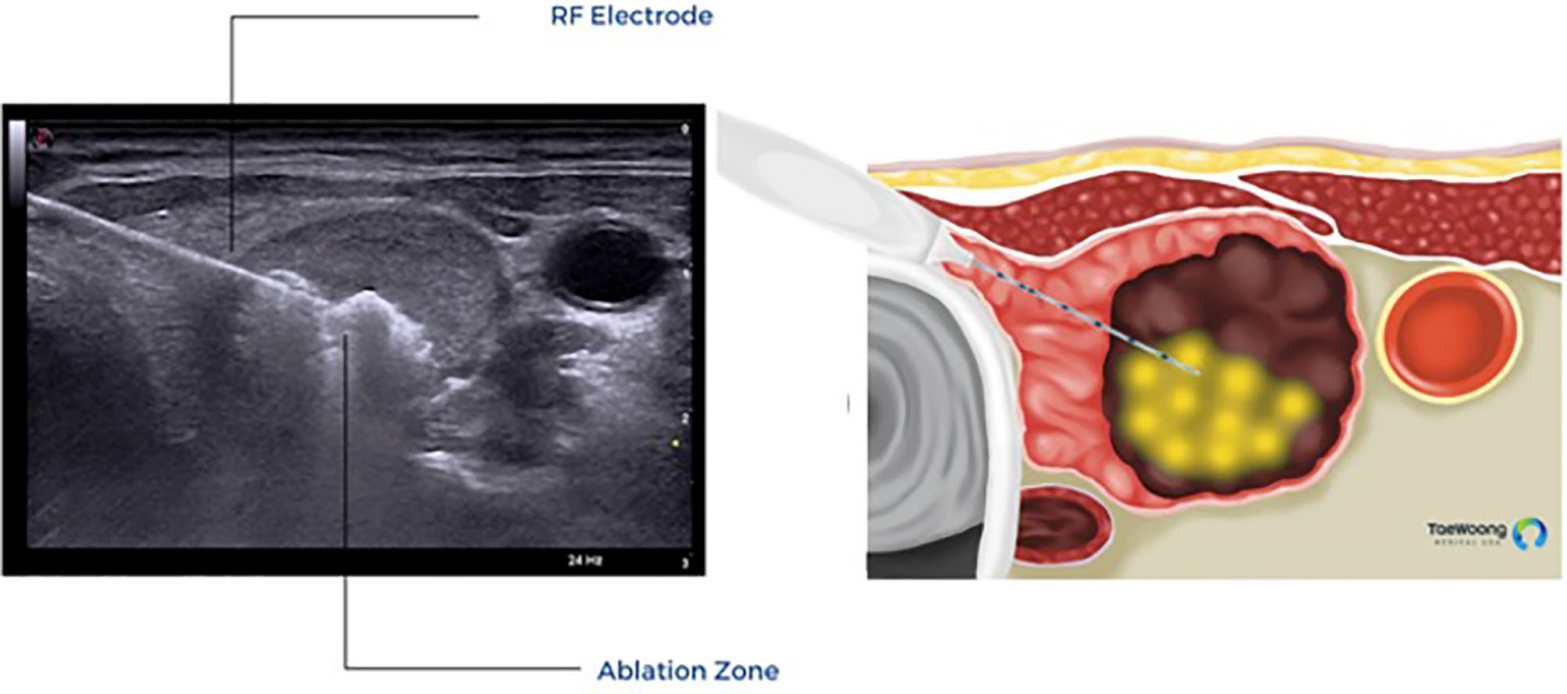
Trans-isthmic approach and moving shot technique used in thermal ablation. (Permission to reprint from Taewoong Medical USA).

The vast majority of these thermal ablation procedures are performed under local anesthesia, which precludes the use of current, FDA approved IONM systems in the United States. IONM for thermal ablation procedures is not only logistically difficult, it is also not essential if providers use appropriate techniques to reduce the risk of thermal injury. Excellent understanding of the sonographic anatomy of the neck, including potential aberrant anatomy such as a medially positioned vagus nerve, is required and the concept of a ‘danger triangle’ adjacent to the posteromedial thyroid capsule has been proposed (**Figure 3**). It is generally recommended to leave a cuff of unablated thyroid tissue immediately adjacent to this region (21, 22, 28, 29). Widely adopted technical concepts such as the trans-isthmic approach and moving-shot technique are advocated in order to decrease the risk of thermal injury (**Figure 4**). Another strategy utilized to decrease the risk of nerve injury is hydrodissection, specifically if the nodule abuts the lateral capsule and the vagus nerve takes an aberrant medial position. Hydrodissection involves a pre-ablation injection of 5% dextrose to create space between the lateral thyroid and the medial vagus to allow for ablation of the lateral most part of the nodule (30). In addition, having the patient speak during the course of the procedure may alert the interventionalist of a possible injury if a change in the quality of the voice is detected. However, despite these precautions, injury to the laryngeal nerves by virtue of their proximity to the target tissue remains a real risk.

## Summary of IONM Studies and Thermal Ablation

In 2021, Sinclair et al. conducted a prospective single institution study to evaluate whether the recurrent laryngeal nerve was affected during RFA of thyroid nodules using continuous intraoperative nerve monitoring (30). Inclusion criteria consisted of symptomatic thyroid nodules with elevated cosmetic and symptom scores and two fine needle aspiration biopsies that demonstrated Bethesda II cytology. All patients underwent a pre procedure laryngoscopy. This was followed by induction of general anesthesia with placement of a monitored electromyography endotracheal tube (NIM EMG-ETT). An electrical stimuli consisting of 1-3 rectangular pulses at a 3-20 mA intensity with a 1-2 ms interstimulus interval and duration of 0.5-1ms was applied to the laryngeal mucosa through electrodes on the ETT contralateral to the RLN at risk to elicit a laryngeal adductor reflex (LAR). The LAR was then recorded by the electrodes on the ipsilateral side of the RLN at risk. All posterior nodules were treated with a max of 40 W and the amplitude was continuously monitored throughout the ablation procedure. The surgeon was careful to not breach the posterior capsule of the thyroid and was notified if there was a decrease >50% from baseline, at which point the procedure was stopped to allow amplitude to recover. Patients then underwent a post procedure laryngoscopy.

A total of 10 patients participated in the study consisting of 20 nodules total. There was no significant change in the LAR during the ablation (no patient experienced a >50% decline in LAR amplitude), no ablation needed to be halted to allow for nerve recovery, and pre and post procedure laryngoscopies were similar. The largest decrease in amplitude was 25% in one patient, which recovered rapidly upon repositioning the electrode using the moving shot technique. Based on this study, it is difficult to determine whether the advantages of continuous nerve monitoring with open surgery (providing feedback of possible impending injury) would also be advantageous in RFA or whether the moving shot technique is protective on its own. The authors concluded that RFA was safe as long as the posterior capsule was not breached by the ablation zone and the power was less than 40 W.

Given that most RFA procedures are performed using local anesthesia, alternative forms of nerve monitoring that do not require the placement of an endotracheal tube have also been investigated. Lin et al. conducted a retrospective cohort study of patients who underwent ultrasound- guided RFA of thyroid nodules between 2/2019 to 8/2019 using neuromonitoring with cutaneous electrodes (31). Inclusion criteria consisted of having a symptomatic nodule (discomfort, compressive symptoms, foreign body sensation), benign cytology on fine-needle aspiration, no BRAF mutation, normal thyroid function tests, and normal movement of the vocal cords on laryngoscopy. The primary endpoint of the study was to determine the feasibility of using neuromonitoring with cutaneous surface electrodes during ultrasound guided RFA of thyroid nodules, which was defined as the ability to obtain nerve stimulation. The secondary end point was achievement of technical success, defined as correlation between the final EMG signal and post procedure laryngoscopy results.

For nerve monitoring, Ambu Neuroline 715 single-patient surface electrodes were placed on the skin anterior to the left and right laminas of the thyroid cartilage (recording electrodes) and on the upper arm (grounding electrodes). A 22-gauge nerve stimulation needle (Stimuplex D; B. Braun, Melsungen, Germany) was attached to a saline syringe and set at 100ms and 4Hz to be used as both a stimulation probe and for hydrodissection. The NIM Nerve monitoring System version 3.0 (Medtronic, Minneapolis, MN, ISA) was used to record electromyograms (EMGs) with an event threshold of 100 μV.

The nerve stimulation needle was inserted under ultrasound guidance between the common carotid artery and the internal jugular vein at the level of the lower pole of the thyroid to stimulate the vagus nerve using a 3.0 mA current (“V1”). A robust initial EMG goal was defined as obtaining an amplitude >500 μV. Once the vagus nerve was identified, hydrodissection with normal saline was performed between the carotid sheath and thyroid gland. The stimulation probe was used to detect the course of the laryngeal nerve along the posterior aspect of the thyroid. If no signal was detected, normal saline was injected to create a plane between the posterior thyroid and the RLN. RFA was performed with a power ranging between 30-50 W. After ablation, the nerve stimulation needle was again inserted into the carotid sheath to obtain a final vagus EMG (“V2”) with a 3.0 mA current. The final EMG (V2) was categorized as normative baseline (absolute amplitude of the signal is not <50% and the growth of its latency is <10% of the initial baseline), impending adverse EMG (amplitude decrease >50% and a latency increase 10% of the initial baseline), or loss of signal (normal vocal cord movement on pre procedure laryngoscopy with a subsequent low (<100 μV) or absent EMG response). For either the impending adverse EMG or loss of signal events, cold liquid was injected posterior to the thyroid gland and into the tracheoesophageal groove and stimulations were performed every 5 minutes to monitor for recovery, which was defined as reaching an absolute amplitude of >50% of the baseline. Patients underwent post procedure laryngoscopy one day after the procedure and RLN injury was confirmed if there was abnormal vocal cord activity. Patients had 6 month follow up with a 10 cm visual analog scale, cosmetic score, and calculation of the volume reduction ratio (VRR).

A total of 16 patients satisfied inclusion and exclusion criteria consisting of 20 nodules. Nerve stimulation was achieved before and after the procedure for all 20 vagus nerves among the 16 patients and 16 of the 20 nerves had a robust initial EMG (>500 μV). There was a significant difference in the mean response amplitude between V1 (612.7 +/- 130 μV) and V2 (592.7 +/- 127.3 μV), p<0.05. However, there was no significant difference between the response latencies of V1 and V2. No patients were categorized as impending adverse EMG or loss of signal. All 20 nerves had a post EMG that correlated with the post laryngoscopy results and there were no instances of injury to the recurrent laryngeal nerve.

At the 6 month follow up, the maximum nodule lesion size decreased from 2.49 +/- 0.73 cm to 1.47 +/- 0.44 cm (p<0.05) and the VRR was 68.5 +/- 21.5%. Furthermore, the mean symptom score decreased from 3.6 +/- 0.7 to 1.2 +/- 0.4 (p<0.05) and the mean cosmetic score decreased from 3.4 +/- 0.5 to 1.6 +/- 0.6 (p<0.05). There were no complications related to nerve monitoring or RFA including hematoma, tracheal or esophageal injury, skin burn, or lidocaine toxicity.

The authors concluded that using cutaneous electrodes for RLN monitoring during RFA was feasible and safe. However, the authors also recognize the limitations of this study including the retrospective nature, small sample size, lack of a control group and the ability of this technique to only provide intermittent nerve monitoring (before and after the procedure). Furthermore, similar to the study by Sinclair, et al, the use of the nerve monitor among these 16 patients did not alter the procedure to prevent a potential nerve injury. Due to the low rate of nerve injury, detecting whether nerve monitoring significantly impacts the rate of nerve injury for RFA will require a much larger sample size than prior studies.

## Conclusion

Intraoperative nerve monitoring has been widely accepted as a useful adjunct to visual identification of the nerve during thyroidectomy. However, most IONM systems require the use of an endotracheal tube to allow for continuous or intermittent nerve monitoring. Such nerve monitoring systems can be used to perform thermal ablation procedures such as RFA, however the procedure must be done under general anesthesia. Consequently, there is a role for nerve monitoring systems that employ cutaneous surface electrodes, allowing for the RFA procedure to be done under local anesthesia. Similar to surgery, protection of the recurrent laryngeal and vagus nerves during RFA requires a thorough understanding of neck anatomy and the ability to infer the course of the RLN and vagus nerves using sonography.

## Author Contributions

All authors listed have made a substantial, direct, and intellectual contribution to the work, and approved it for publication.

## Conflict of Interest

The authors declare that the research was conducted in the absence of any commercial or financial relationships that could be construed as a potential conflict of interest.

## Publisher’s Note

All claims expressed in this article are solely those of the authors and do not necessarily represent those of their affiliated organizations, or those of the publisher, the editors and the reviewers. Any product that may be evaluated in this article, or claim that may be made by its manufacturer, is not guaranteed or endorsed by the publisher.
